# Supplementary material for: Construction and Validation of a Novel Prognosis Model in Colon Cancer Based on Cuproptosis-Related Long Non-Coding RNAs
Source: J Clin Med. 2023 Feb 15;12(4):1528. doi: 10.3390/jcm12041528 (PMC9960235; doi:10.3390/jcm12041528)
Supplement: Supplementary file 1 [file jcm-12-01528-s001.zip › Supplementary Figure and Table Legends.pdf]

Supplementary Material

Supplementary Figure S1. Principle Component Analysis (PCA) of Samples in CC Dataset. (A) PCA plot for the distribution of mRNAs in CC patients with high and low CRLs-risk score. (B) PCA plot demonstrating the distribution of cuproptosis-related mRNAs in CC patients with high and low CRLs-risk score. (C) PCA plot depicting the distribution of CRLs in CC patients with high and low CRLs-risk score. (D) PCA plot illustrating the distribution of prognosis-associated CRLs in CC patients with high and low CRLs-risk score.

Supplementary Table S1. Expression profile of CRGs in TCGA-COAD.

Supplementary Table S2. Primers of cuproptosis-related lncRNAs used for qPCR in this study.

Supplementary Table S3. CRLs-risk score of cell lines and patients' samples
