# Supplementary figures and images for: Construction and Validation of a Novel Prognosis Model in Colon Cancer Based on Cuproptosis-Related Long Non-Coding RNAs
Source: J Clin Med. 2023 Feb 15;12(4):1528. doi: 10.3390/jcm12041528 (PMC9960235; doi:10.3390/jcm12041528)

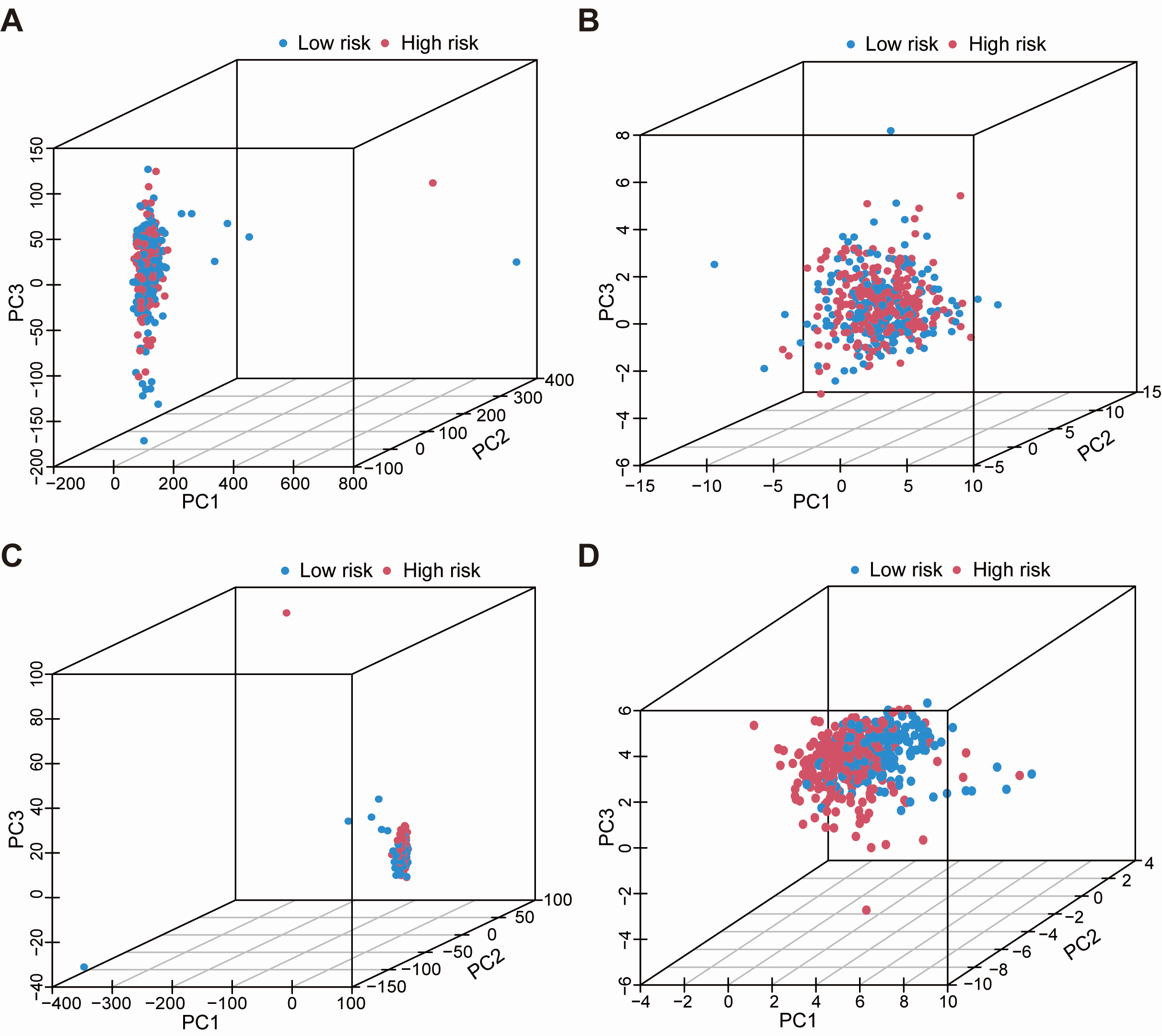

Supplement: Supplementary file 1 [file jcm-12-01528-s001.zip › Supplement Figure S1.tif]
